# Supplementary material for: Improved Device Distribution in High-Performance SiNx Resistive Random Access Memory via Arsenic Ion Implantation
Source: Nanomaterials (Basel). 2021 May 25;11(6):1401. doi: 10.3390/nano11061401 (PMC8226572; doi:10.3390/nano11061401)
Supplement: Supplementary file 1 [file nanomaterials-11-01401-s001.zip › nanomaterials-1217499-supplementary.pdf]

nanomaterials

Supplementary Materials

Improved Device Distribution in High Performance SiN_x Resistive Random Access Memory via Ion Implantation

Te Jui Yen[1], Albert Chin [1,*] and Vladimir Gritsenko [2,3,4]

[1] Department of Electronics Engineering, National Yang Ming Chiao Tung University, Hsinchu 300, Taiwan
[2] Rzhanov Institute of Semiconductor Physics, Siberian Branch, Russian Academy of Sciences, Novosibirsk, Russia
[3] Novosibirsk State University, Novosobirsk, Russia
[4] Novosibirsk State Technical University, Novosibirsk, Russia
* Correspondence: email: achin@nycu.edu.tw; Tel.: +886-3-5731841

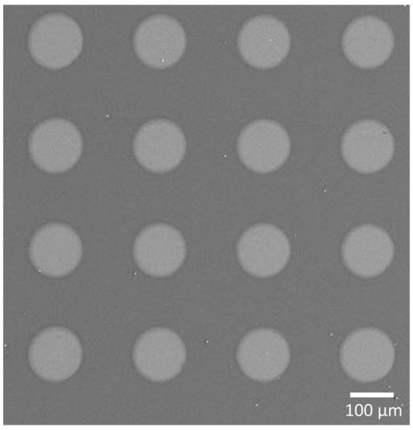

(**a**)

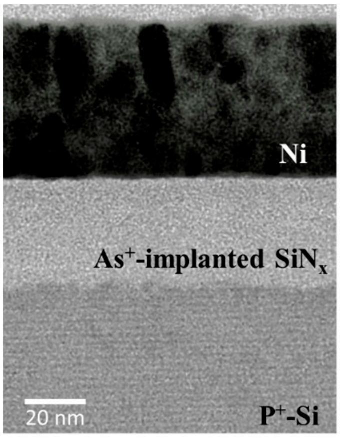

(**b**)

Figure S1. (**a**) Top-view SEM and (**b**) cross-sectional TEM image of the SiN_x RRAM device.

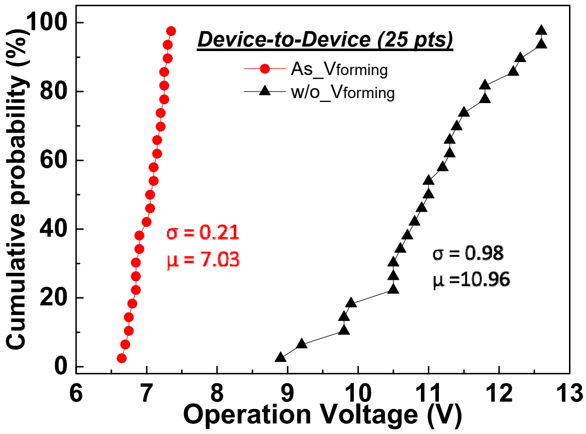

Figure S2. Forming voltage distributions of As^+-implanted and nonimplanted SiN_x RRAM devices.

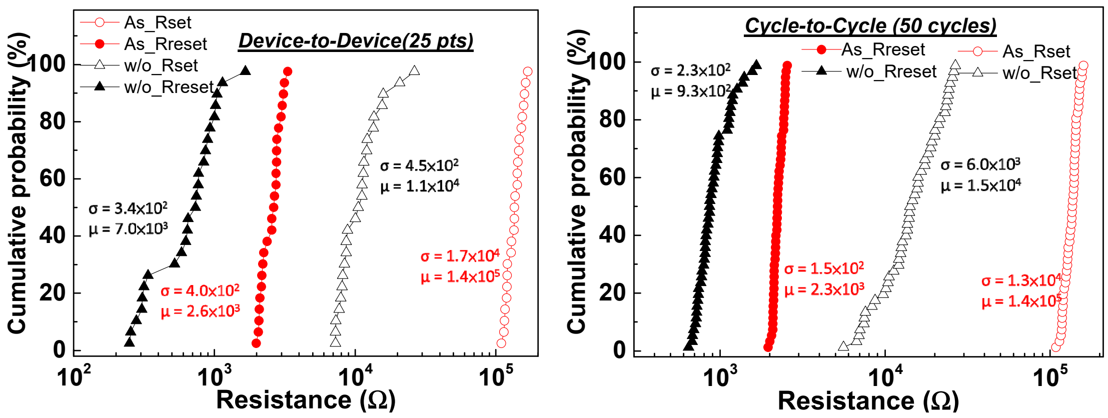

(**a**) (**b**)

Figure S3. The (**a**) device-to-device and (**b**) cycle-to-cycle of R_{set}-R_{reset} distributions of the As⁺-implanted and nonimplanted SiN_x RRAM devices.

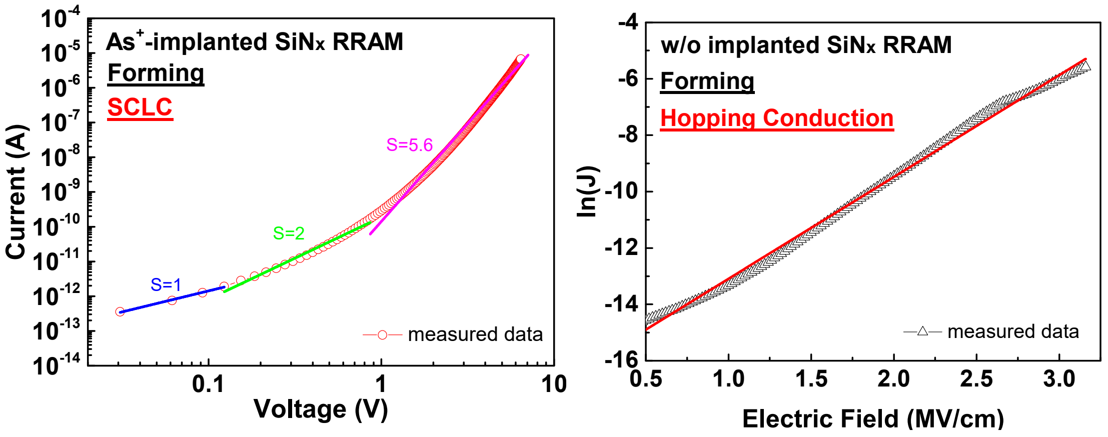

(**a**) (**b**)

Figure S4. The analyzed *I-V* curves of (**a**) As⁺-implanted and (**b**) nonimplanted SiN_x RRAM devices before forming process.
